# Inhibitory Effects of *Trypanosoma cruzi* Sialoglycoproteins on CD4^+^ T Cells Are Associated with Increased Susceptibility to Infection

**DOI:** 10.1371/journal.pone.0077568

**Published:** 2013-10-28

**Authors:** Marise Pinheiro Nunes, Bárbara Fortes, João Luiz Silva-Filho, Eugênia Terra-Granado, Leonardo Santos, Luciana Conde, Isadora de Araújo Oliveira, Leonardo Freire-de-Lima, Marina Vieira Martins, Ana Acacia Sá Pinheiro, Christina Maeda Takyia, Célio Geraldo Freire-de-Lima, Adriane Regina Todeschini, George Alexandre DosReis, Alexandre Morrot

**Affiliations:** 1 Oswaldo Cruz Foundation, Rio de Janeiro, Brazil; 2 Institute of Microbiology, Federal University of Rio de Janeiro, Rio de Janeiro, Brazil; 3 Carlos Chagas Filho Institute of Biophysics, Federal University of Rio de Janeiro, Rio de Janeiro, Brazil; 4 National Cancer Institute, Rio de Janeiro, Brazil; Albert Einstein College of Medicine, United States of America

## Abstract

**Background:**

The *Trypanosoma cruzi* infection is associated with severe T cell unresponsiveness to antigens and mitogens characterized by decreased IL-2 synthesis. *Trypanosoma cruzi* mucin (Tc Muc) has been implicated in this phenomenom. These molecules contain a unique type of glycosylation consisting of several sialylated *O*-glycans linked to the protein backbone via N-acetylglucosamine residues.

**Methodology/Principal Findings:**

In this study, we evaluated the ability of Tc Muc to modulate the activation of CD4^+^ T cells. Our data show that cross-linking of CD3 on naïve CD4^+^ T cells in the presence of Tc Muc resulted in the inhibition of both cytokine secretion and proliferation. We further show that the sialylated O-Linked Glycan residues from tc mucin potentiate the suppression of T cell response by inducing G1-phase cell cycle arrest associated with upregulation of mitogen inhibitor p27^kip1^. These inhibitory effects cannot be reversed by the addition of exogenous IL-2, rendering CD4^+^ T cells anergic when activated by TCR triggering. Additionally, *in vivo* administration of Tc Muc during *T. cruzi* infection enhanced parasitemia and aggravated heart damage. Analysis of recall responses during infection showed lower frequencies of IFN-γ producing CD4^+^ T cells in the spleen of Tc Muc treated mice, compared to untreated controls.

**Conclusions/Significance:**

Our results indicate that Tc Muc mediates inhibitory efects on CD4^+^ T expansion and cytokine production, by blocking cell cycle progression in the G1 phase. We propose that the sialyl motif of Tc Muc is able to interact with sialic acid-binding Ig-like lectins (Siglecs) on CD4^+^ T cells, which may allow the parasite to modulate the immune system.

## Introduction

Chagas disease is caused by the protozoan parasite *Trypanosoma cruzi* and is an important endemic infection in Latin America. Lately, it has also become a health concern in the United States, Canada and Europe [1], [2]. The parasite is transmitted via the faeces of insect vectors of the family Reduviidae [3]. When the parasite enters the host it evokes a strong immunological response that is able to control the parasitic multiplication but not eliminate it [4]–[6]. After a delay that can be as much as 20 years, about a third of infected patients enter the chronic phase, characterized by the symptoms of Chagas disease [7] It is not yet clear how the observed pathology is triggered, but there is considerable evidence that persistence of the parasite is associated with a chronic inflammatory response, a major cause of Chagas disease [8]–[13].

*T. cruzi* employs a variety of strategies to evade the immune system and maintain itself in the infected host. The main method involves inhibiting specific T-cell responses so that it frequently establishes chronic infections [12]–[19]. A number of both host-dependent and parasite-induced mechanisms accomplish this immune regulation [20]. The T cells of infected hosts are largely unresponsive to antigens and mitogens, and this results in reduced IL-2 synthesis and increased nitric oxide (NO) production. Although spleen cell responses to ConA were more apparent in infected IFN-γR^−/−^ or inducible nitric oxide synthase (iNOS)-deficient mice than in their control littermates, IL-2 production remained as strongly affected [14].

It is thought that the large number of *O*-glycosylated Thr/Ser/Pro-rich mucin molecules (Tc Muc) on the surface of *T cruzi* are the main acceptors of sialic acid and are shown to be responsible for most of the immune effects of infection [14]–, [21]–[26]. *T. cruzi* cannot synthesize sialic acid but it produces a surface *trans*-sialidase that transfers sialic acid from the sialoglycoconjugates of the host to the parasite glycoconjugates, especially to terminal β-galactosyl residues of Tc Muc [27]–[30]. Sialylated glycoconjugates are believed to play a role in a number of host–parasite interactions, such macrophage attachment, avoidance of complement lysis, and alteration of host immune responses [28], [31]–[36]. *T. cruzi* mucin has been shown to inhibit T cell proliferation as well as IL-2 production and transcription in response to mitogens and to anti-CD3. This effect involves action at the transcriptional level, since Tc Muc inhibits transcription driven from the IL-2 promoter [15], [16]. Moreover, transcription of reporter genes under the control of CD28RE, NFAT and AP-1, but not of NF-κB sites, is also inhibited by Tc Muc to different extents, with the greatest effect being on NFAT. In agreement with this, overexpressing NFAT markedly reduced Tc Muc inhibition of IL-2 transcription. Tc Muc also inhibits early events in T cell activation such as tyrosine phosphorylation of the adapter protein SLP-76 and the tyrosine kinase ZAP-70 [14].

Although sialylated glycoconjugates play important roles in the initiation, persistence, and pathogenesis of Chagas’ disease, their precise roles and their host receptors remain unknown. There is evidence that sialylated Tc Muc can interact with Siglec-E (CD33), a member of the Siglec family of sialic acid-binding Ig-like lectins found mainly on cells of the immune system [33], [37]. Siglecs have immunoreceptor tyrosine-based inhibitory motifs (ITIMs) in their cytosolic tails, which suggests that they are able to perform inhibitory function when they bind sialylated carbohydrates [38]–[40]. Siglec-E is a restricted leukocyte antigen mainly expressed on mouse phagocytic cells and on antigen-presenting cells (APCs) including macrophages and dendritic cells [41], [42]. The binding of pathogenic *T. cruzi* to Siglec-E-expressing cells is followed by rapid mobilization of Siglec-E into the contact zone between parasite and host cells. It appears that binding of Siglec-E affects the activity of APCs, leading to lower production of IL-12, which is important for Th1 responses [33], [37].

The present study shows that cross-linking of CD3 on naïve CD4^+^ T cells in the presence of Tc Muc resulted in the inhibition of both cytokine secretion and lymphoproliferative response as compared to the controls obtained upon TCR triggering. The *T. cruzi* mucin-induced suppression of CD4^+^ T cell response is mediated by G1 cell cycle arrest and is associated with up-regulation of the cyclin-dependent kinase inhibitors p27^kip1^. Interestingly, i*n vivo* administration of Tc Muc during murine experimental infection with *Trypanosoma cruzi* parasites rendered lower frequencies of splenic IFN-γ producing CD4^+^ T cells in the host compared to infected controls. These effects were accompanied by a greater susceptibility to infection, as shown by higher levels of parasitemia in infected mice treated with Tc Muc compared to non-treated infected controls. In the present work, we support evidence that sialylated O-Linked Glycan residues of Tc Muc exert inhibitory effects on CD4^+^ T cells through the interaction of the sialyl motif with the sialic acid-binding Ig-like lectin host receptors (Siglecs). We propose that signaling of CD4^+^ T cells via Siglecs is at least in part responsible for the induction of T cell anergy, and that this may allow the parasite to interfere with the host immune system.

## Materials and Methods

### Ethics Statement

This work was approved by the Research Ethics Committee of Fiocruz (protocol CEUA-LW8/10). Protocols for animal studies were approved by the Institutional Ethical Committees in accordance with international guidelines. All animal experimentation was performed in accordance with the terms of the Brazilian guidelines for the animal welfare regulations.

### Preparation of Sialogycoproteins from *T. cruzi* DM28c Strain

Sialoglycoproteins from *T. cruzi* DM28c were obtained as described (Agrellos et al., 2003). Epimastigote forms were grown in 1 l of brain heart infusion medium containing 10% fetal calf serum, and supplemented with 10 mg/l hemin and 20 mg/l folic acid. Cultures were incubated at 28°C with shaking (100 rpm) for 5–7 days. Cells were harvested by centrifugation, washed three times with 0.9% NaCl and frozen at −20°C. Frozen cells were thawed, extracted with cold water and the pellet recovered by centrifugation for three times. The pellet was, than extracted with 45% (v/v) aqueous phenol at 75°C. The aqueous phase of the phenol extract was dialyzed, lyophilised, redissolved in water, and applied into a Bio-Gel P-60 column. Carbohydrate-containing material in the excluded volume was lyophilized and suspend in chloroform/methanol/water (10∶10∶3 v/v). The glycoproteins in this solvent mixture were lyophilized and applied into an octyl-sepharose column and eluted with 60% (v/v) propanol in water. The mucin obtained were analyzed by sodium dodecyl sulfate (SDS)-polyacrylamide gel electrophoresis and stained with periodic acid/Schiff’s reagents for carbohydrate detection. In order to obtain a lipopolysaccharide (LPS)-free preparation the sialoglycoproteins obtained were passed through an agarose-immobilized polymyxin B column (Sigma Chemical Co., MO). For desialylation reaction, purified mucin was subjected to treatment with 0.2 U/ml of *Vibrio cholerae* neuraminidase, in PBS pH 6.0 containing 1 mM CaCl_2_. After incubation at 37°C for 1 h, the enzyme was heat-inactivated and the solution was applied into an octyl-sepharose column. The desialylated mucin was eluted with 60% (v/v) propanol in water. The eluted sample was dried by rotatory evaporation, resuspended in water and lyophilized.

### Animals, Infection and *In vivo* Tc Muc Treatment

Male BALB/c mice, aged 6–8 weeks, were obtained from the Oswaldo Cruz Foundation animal facility. Epimastigotes of *T. cruzi* Dm28c clone were cultured at 27°C in Bacto™ Brain Heart Infusion (BHI, Becton Dickinson Company, USA) supplemented 10 µg/mL hemin, 0,02 g/L folic acid (both from Sigma-Aldrich, USA) and 10% of heat inactivated fetal bovine serum (FCS, Gibco/Lifetechnologies). Acute infection was performed by inoculating the animals intraperitoneally with 2×10^5^ chemically induced metacyclic forms of *Trypanosoma cruzi* Dm28c clone obtained as described [43]. *T. cruzi* mucin diluted in PBS was administered via I.P. at 20 µg/mouse and on alternate days starting at day of infection until day 22 after infection and sacrificed on day 24. A control group was treated with PBS using the same regimen. Parasitemia was monitored on days 7, 9, 11, 13, 15, 18, 20 and 22 post infection in blood obtained from tail vein and lysed in Tris-buffered ammonium chloride by counting trypomastigotes forms. Mice were killed during the acute phase, at 24 days post infection.

### T Cell Purification and *in vitro* Proliferation

Primary T-cell-enriched populations from naïve mice were obtained by nylon wool filtration of unfractionated splenic cell suspensions previously depleted of erythrocytes by treatment with Tris-buffered ammonium chloride. Highly purified CD4^+^ T cells were nonadherent cell treated with anti-CD8, anti-B220, anti-MHC class II, anti-MAC-1, anti-αβTCR (all at 10 µg/mL, BD Pharmingem™) and purified with anti-IgG-coated magnetic beads (Biomag perseptive Biosystems). CD4^+^ T cells were cultured in DMEM supplemented with 2 mM glutamine, 5×10^−5^ M 2-ME, 10 µg/mL gentamicin, 1 mM sodium pyruvate, and 0.1 mM MEM nonessential amino acids (all from Gibco™, Invitrogen Corporation) plus 1% Nutridoma-SP (Roche, Germany) instead of FBS. For proliferation assays, CD4^+^ T cells (3×10^5^ cells/well) were re-suspended in complete culture medium containing 1% Nutridoma and were stimulated with plate bound anti-CD3 mAb (5 µg/mL, clone 145-2C11, BD Pharmingen), with or without *T. cruzi* mucin or control mucin in 96-well flat bottom plates. For the dose response experiments, CD4^+^ T cells (3×10^5^ cells/well) stimulated or not with plate bound anti-CD3 (5 µg/mL) were cultured with different concentrations of TcMuc (10, 20 or 50 µg/mL). In some experiments IL-2 (10 U/mL) (BD-Pharmingen) were added at the beginning of the culture period to wells containing CD4^+^ T cells stimulated with plate bound anti-CD3 in the presence of Tc Muc (20 µg/mL). CD4^+^ T cells cultures were also performed with desialylated Tc Muc (20 µg/mL). Anti-CD33 or isotype control (40 µg/mL, R&D Systems) were added to CD4^+^ T cells (3×10^5^ cells/well) cultivated in 96-well flat bottom plates coated with anti-CD3. Cultures were incubated 3 days at 37°C and 7% CO_2_ in a humid atmosphere and pulsed with 1 µCi of tritiated thymidine ([^3^H]TdR, 5,0 Ci/mmol, Amershan) 16 hours before harvested. Thymidine incorporation was determined in a scintillation counter (Beckman Coulter™ - LS 6500 Multipurpose Scintillation Counter). Results shown were mean and SE of cultures done in triplicates. Cell viability was assessed using the metabolic assay MTT, as previously described [44].

### T Cell Activation and Cytokine Assays

For restimuation assay, CD4^+^ T cells (1×10^6^/0,5 mL) were cultured in 48 well plate with medium only, stimulated with 5 µg/mL plate-bound anti-CD3 in the presence or absence of *T. cruzi* mucin (Tc Muc, 20 µg/mL) or control mucin (Ct Muc, Mucin Type I from bovine sub maxillary glands, 20 µg/mL, Sigma-Aldrich). Before adding CD4^+^ T cells to plates, wells were pre-washed 3 times by adding cold HBSS (Gibco, Invitrogen) to remove excess antibody. Plates were incubated at 37°C and 7% CO_2_ in a humid atmosphere. After 3 days of stimulation *in vitro*, cells were harvested, viable cells isolated with Ficoll (1.09 g/ml density, Sigma), washed two times in cold HBSS, counted and 3×10^5^ cells/200 µL were restimulated in flat bottomed 96 well plate (Corning, Costar) coated with anti-CD3 mAb at 5 µg/mL, in the presence or not of Tc Muc (20 µg/mL) or CtMuc (20 µg/mL). Supernatants from those cultures were collected after 48 hours and cytokine levels (IFN-γ, TNF-α, IL-2, IL-4, IL-10 and TGF-β) were assayed by ELISA utilizing purified and biotinylated Abs (R&D Systems), biotin-conjugated streptavidin-alkaline phosphatase (BD Pharmingen™) and developed with ELISA Develpment Kit from R&D System according to the manufacturer’s instructions. Plates were read at 405 nm and values are presented as pg cytokine/mL (mean ± SE). Statistical differences between mean values were evaluated by ANOVA, and pair-wise comparisons were done by the Tukey test.

### Detection of Intracellular Cytokine by Flow Cytometer

Fresh spleen cells were harvested from non-infected or from infected mice at 8 or 15 days post infection (DPI). Cells were washed in PBS (containing 2% fetal bovine serum) and incubated for 30 min at 4°C with anti-CD16/CD32 for Fc blocking. For phenotypic analysis of T cells by FCM, we performed three-color labeling for 30 min at 4°C, using allophycocyanin (APC)-labeled anti-CD4 and fluorescein isothiocyanate (FITC)-labeled anti-CD8 monoclonal antibodies, followed by phycoerythrin (PE)-labeled antibody anti-CD69. All monoclonal antibodies (mAbs) used in FCM were from BD Pharmingen™. Cells were washed and resuspended in PBS supplemented with 2% fetal bovine serum, and data were acquired on a FACSCalibur system (BD Biosciences). Analyses were done after recording 25,000–50,000 events for each sample, using a CELLQuest software (BD Biosciences). To determine the number of IFN-γ-producing T cells in the infected spleen, intracellular cytokine staining was performed. Single cell suspension of infected spleen was prepared, and 10^6^ cells/well were cultured in 96-well U-bottom plates. Cells were left untreated or policlonal stimulated with PMA (20 ng/ml) and ionomycin (500 ng/ml) for 3 h at 37°C in 5% CO2. Brefeldin A (10 µg/ml) was added to the culture for the intracellular cytokine accumulation. Cell surface marker and intracellular cytokine staining for IFN-γ was performed using a Cytofix/Cytoperm kit (BD Pharmingen). All samples were collected with a FACScalibur and were analyzed with Summit 4.3 2 software (Dako).

### Assessment of Cell-cycle Arrest and Western Blotting Analysis

Purified CD4^+^ T cells (2×10^6^ cells/well, 1 mL) were cultured in 24 well plates, stimulated or not with plate bound anti-CD3 (5 µg/mL), in the presence or absence of TcMuc or desialylated Tc Muc (20 µg/mL) for 3 days at 37°C and 7% CO_2_ in a humid atmosphere. At the end of incubation period, cells were fixed with 70% ethanol and stained with propidium iodide (PI, 20 µg/ml, BD Immunocytometry Systems, USA) in PBS containing 0.1% Triton-X-100 and RNAse (10 µg/ml) for 15 min. Data was acquired on a BD FACS Calibur flow cytometer using CellQuest software (BD Immunocytometry Systems, USA). For analysis of the cyclin D3 and p27 expression, cells were alternatively harvested after 3 days of stimulation and lysed in RIPA lysis buffer. Lysates were centrifuged at 16,000×*g* for 10 min at 4°C and the proteins present in the supernatants were solubilized in a SDS sample buffer for electrophoresis by boiling for 5 min and fractionated in SDS-PAGE 9%. The proteins were transferred to PVDF membranes (Trans-Blot system, Bio-Rad) and the membranes were incubated overnight with anti-cyclin D3, anti-p27 and anti-actin (Cell Signaling Technology, Inc.), followed by horseradish peroxidase (HRP)-conjugated anti-mouse secondary antibodies IgG for ECL quimioluminescence reaction (Amersham-Pharmacia).

### Tissue Preparation and Histochemistry

Heart tissues were harvested 24 days after *Trypanosoma cruzi* infection into BALB/c mice. Tissues were fixed in 10% neutral buffered formalin, dehydrated, and paraffin embedded. Sections (4 µm) were obtained and stained with hematoxylin-eosine for topographical analyses of heart tissues. Sections were analyzed under light microscopy. Positive identification of leukocyte infiltration was determined by matching nuclear morphology and cytoplasmic color. Inflammatory score present in the tissue were determined in 40 sequential sections per mouse. Statistical differences between mean values were evaluated by ANOVA, and pairwise comparisons were done by the Tukey test.

### Statistical Analysis

Statistical analyses were performed with GraphPad Prism 4 software, using one-way ANOVA test. Results were expressed as mean ± standard error (S.E.), Differences between control and treated group were considered statistically significant when *P≤*0.05.

## Results

### Tc Muc Supresses CD4^+^ T Cell Proliferation

**Figure 1:**
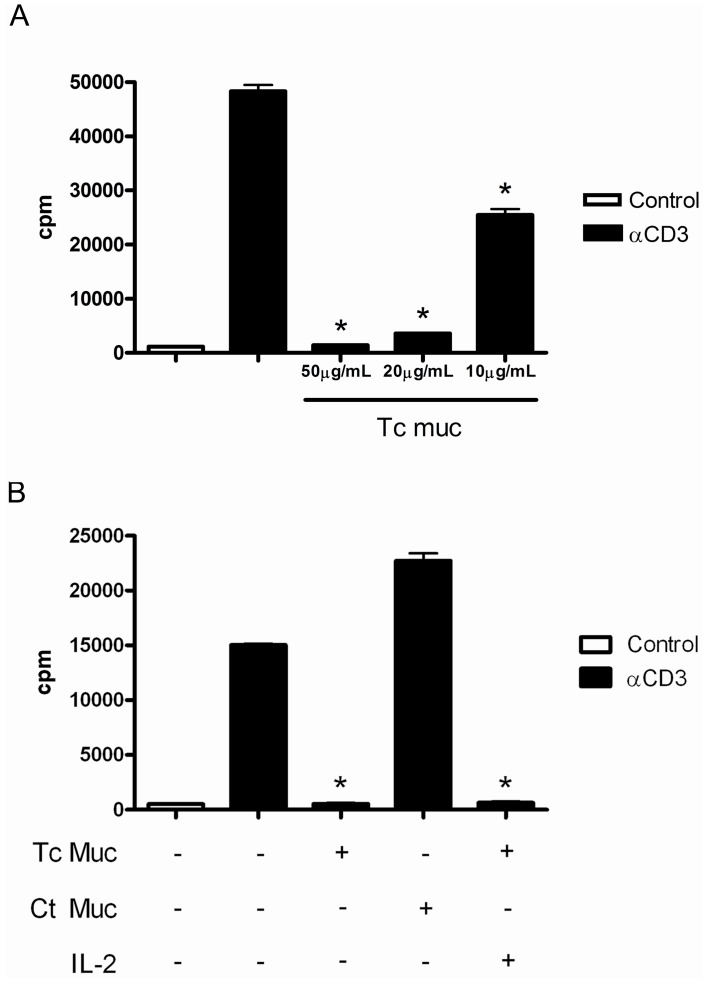
Tc Muc inhibits CD4^+^ T cell proliferation. (**A**) Purified CD4^+^ T cells from naïve spleens were stimulated with pre-coated anti-CD3 for 72 hr, in the presence or absence of increasing concentrations of Tc Muc (10, 20 and 50 µg/mL). Proliferation was measured 72 h after stimulation by [^3^H]thymidine incorporation. (**B**) The inhibition of proliferation by Tc mucin was not observed when control mucin derived from bovine submaxillary glands was used, nor was it reverted by addition of exogenous IL-2 when naïve splenic purified CD4^+^ T cells were stimulated with pre-coated anti-CD3 for 72 hr. Results are the means ±SE of triplicate cultures of three different experiments. *Differences between Tc mucin treatment *versus* anti-CD3 stimulated positive control are significant (*P≤*0.05).

To evaluate the effect of Tc mucin on *in vitro* CD4^+^ T cell activation and proliferation, equal numbers of purified CD4^+^ T cells isolated from naive mice were stimulated by plate bound anti-CD3 mAb in the presence or absence of graded doses of Tc Muc. Our results indicated a significant inhibition of CD4^+^ T cell proliferative response in a dose-reponse manner by Tc Muc, with a marked inhibition after 20 µg/mL (Figure 1a). However, in the control culture, the CD4^+^ T cell population retained the ability to respond to plate bound anti-CD3 mAb, indicating that these cells were fully capable of transmitting activation signals, leading to cell proliferation through the TCR/CD3 receptor (Figures 1a and b). Similar results showing the inhibitory effect of Tc Muc on CD4^+^ T cell activation were obtained upon stimulation by plastic-absorbed anti-Thy1.1 mAb (Figure S1).

**Figure 2:**
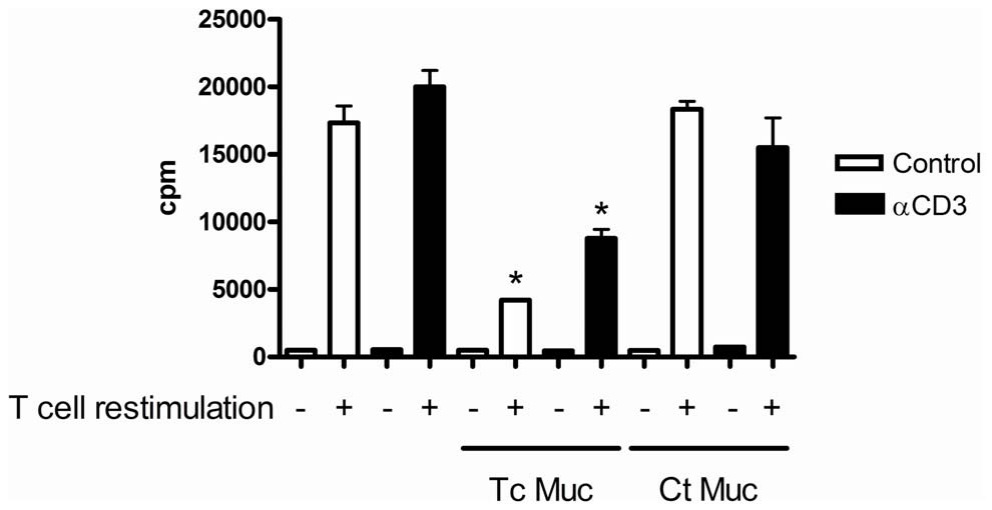
Tc Muc inhibits anti-CD3 restimulation of activated CD4^+^ T cells. Naϊve splenic CD4^+^ T cells were stimulated in 48-well culture plates coated with anti-CD3 (5 µg/mL) in the presence or absence of Tc Muc (20 µg/mL) or the same amount of the bovine mucin as control. After 72 hr of stimulation, activated CD4^+^ T cells were harvested and restimulated for an additional 3 days with plate-bound anti-CD3 in the presence or absence of the TcMuc (20 µg/mL). Proliferation was measured 72 h after stimulation by [^3^H]thymidine incorporation. *Differences between Tc mucin treatment *versus* anti-CD3 stimulated positive control are significant (*P≤*0.05). The data represented above are representative of one of three experiments with similar results.

To determine whether the addition of IL-2 is able to overcome the inhibitory effect of TcMuc, CD4^+^ T cells were stimulated with plate bound anti-CD3 mAb and cultured for 72 hr in the presence of TcMuc and recombinant IL-2 (rIL-2). In these conditions, rIL-2 could not prevent the responsiveness of CD4^+^ T cell to TCR-mediated T cell activation induced by TcMuc (Figure 1b). However, our results indicate that alterations in the patterns of mucin *O*-glycosylation has a possible influence on the inhibitory effect mediated by Tc Muc on CD4^+^ T cells, as this phenomenon was not observed when murine naïve CD4^+^ T cells were cultivated under similar conditions with bovine submaxillary gland mucin (Figure 1b). Furthermore, we showed that when restimulated with anti-CD3, activated CD4^+^ T cells cultured in the presence of Tc Muc was not able to respond to the polyclonal stimulus, indicating that the effect of Tc mucin on T cell mitogen responses bypasses the early receptor signaling of T cell activation (Figure 2).

### Tc Muc Downmodulates Cytokine Expression

**Figure 3:**
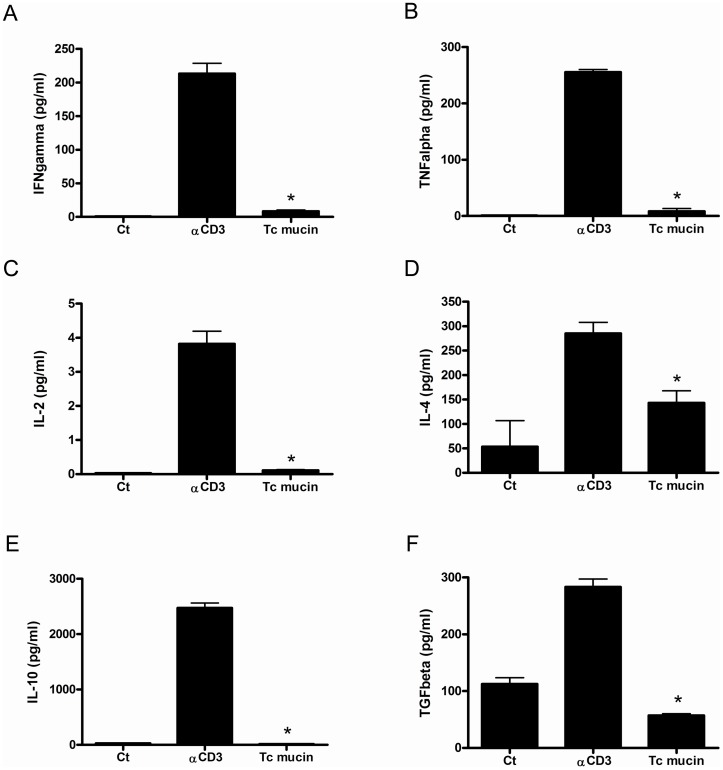
Tc mucin inhibits cytokine production upon TCR stimulation. Purified CD4^+^ T cells from naïve spleens were stimulated with plate bound anti-CD3 (5 µg/mL), in the presence or absence of Tc Muc (20 µg/mL). Cytokines IL-2, IL-4, IL-10, IFN-γ, TNF-α and TGF-β were detected by ELISA in the supernatants obtained after 48 h stimulation. All cytokine values in the presence of Tc Muc were significantly lower than controls (*P≤*0.05). Results are the means ±SD of triplicate cultures of three different experiments.

Our data showing that Tc Muc promotes unresponsiveness of CD4**^+^** T cells upon mitogen activation led us to investigate the cytokine profile of these cells. To address this question, purified CD4**^+^** T cells isolated from naive mice were stimulated by plate-bound anti-CD3 mAb in the presence or absence of Tc Muc for 2 days, then the supernatants were collected and tested for the cytokines IFN-γ, TNF-α, IL-2, IL-4, IL-10 and TGF-β. Our results demonstrate that, at day 3, Tc mucin treatment of activated cells resulted in a significant decrease in all the cytokines analysed when compared to the anti-CD3 stimulated positive control (Figure 3). The suppressive effect of Tc Muc on CD4^+^ T cell activation and expansion *in vitro* is not correlated with any possible effect of the mucin on the death induction of CD4**^+^** T cells, since viability was not significantly affected after 3 days in culture as evaluated by MTT cell viability assay (Figure S2).

### Inhibition of CD4^+^ T Cell Proliferation is Partially Reverted upon Neuraminidase-treatment of *T. cruzi* mucin and is Associated to Upregulation of p27^Kip1^

**Figure 4:**
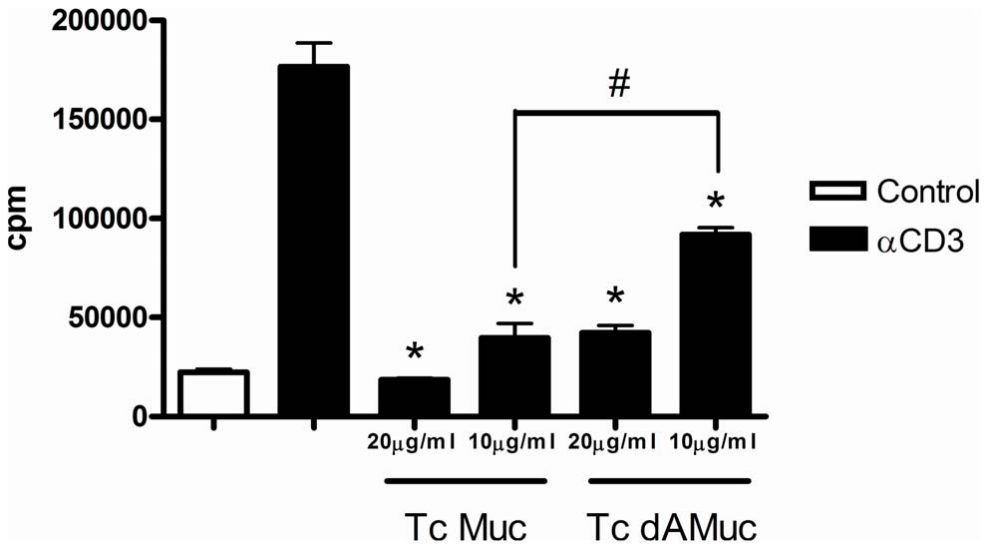
Inhibition of CD4^+^ T cell proliferation is partially recovered upon neuraminidase-treatment of *T. cruzi* mucin. Purified CD4^+^ T cells from naïve spleens were stimulated with plate bound anti-CD3 for 72 hr, in the presence or absence of increasing concentrations of native or desialylated Tc Muc (10 and 20 µg/mL). Proliferation was measured 72 h after stimulation by [^3^H]thymidine incorporation. *Differences between native or desialylated Tc Muc treatment *versus* anti-CD3 stimulated positive controls are significant (*P≤*0.05). #The inhibition of proliferation by Tc Muc was partially recovered when *T. cruzi* mucin was desialylated by previous treatment with neuraminidase (*P* = 0.0023). Results are the means ±SE of triplicate cultures. This experiment was repeated three times, with similar results each time.

The sialic acid residues are incorporated into Tc Muc (Figure S3 and S4) in a reaction catalyzed by the parasite *trans*-sialidase [27]–[30]. This sialylation influences the effectiveness of the inhibitory properties of Tc Muc on dendritic cell function through the interaction with the sialic acid-binding Ig-like lectins that are predominantly expressed on cells of the immune system [33], [37]. We next investigated the role of terminal sialic acid in CD4^+^ T cell activation. For this purpose, Tc Muc was subjected to treatment with 0.2 U/mL of *V*. *cholerae* neuraminidase to remove the sialic acid terminal residues. To this end, purified CD4^+^ T cells from naïve spleen were stimulated with plate-bound anti-CD3 for 72 hr, in the presence or absence of increasing concentrations of native or desialylated Tc Muc (10 and 20 µg/ml), and proliferation was measured 72 h after stimulation as [^3^H]thymidine incorporation. Our findings demonstrated that the inhibition of proliferation by Tc Muc was partially reverted when *T. cruzi* mucin was desialylated by previous treatment with neuraminidase, indicating a possible role for the sialic acid-binding Ig-like lectins’ receptors in the inhibitory effects on CD4^+^ T cells (Figure 4).

**Figure 5:**
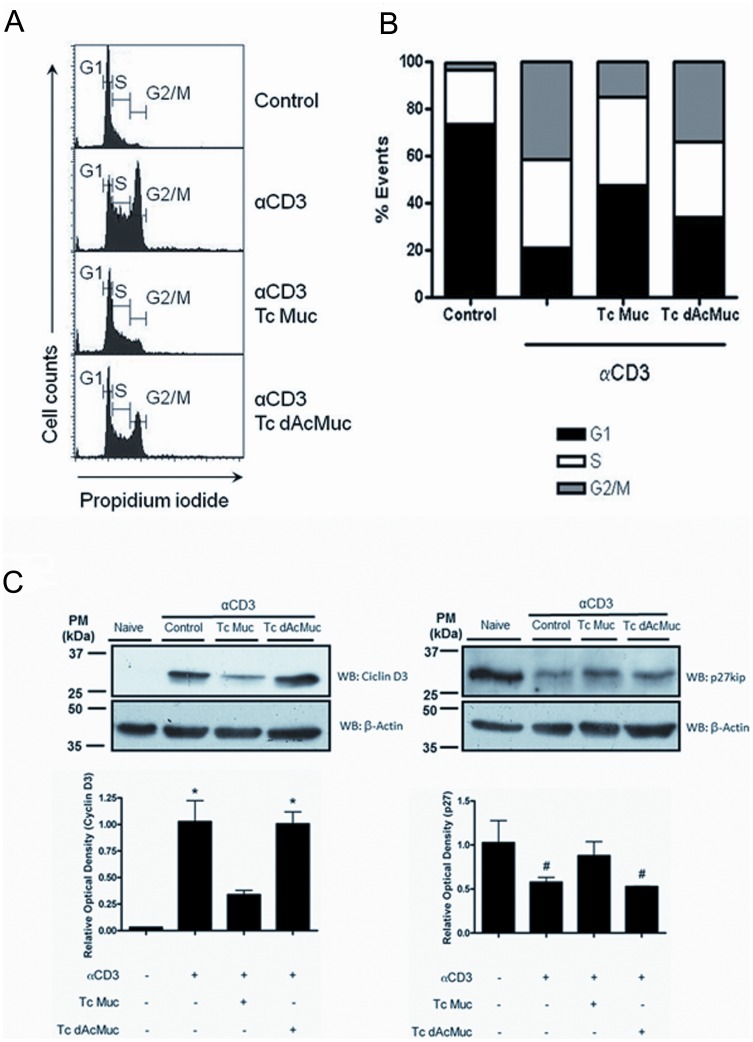
Tc Muc induced G1 cell cycle arrest of CD4^+^ T cells correlates with upregulation of Cyclin D3 and downregulation of p27^kip1^. Purified CD4^+^ T cells from naïve spleens were stimulated with plate bound anti-CD3 in the presence or absence of native or desialylated *T. cruzi* mucins (20 µg/ml). After 3 days, cell cycle analysis (A) was evaluated by flow cytometry based on propidium iodide (PI) intercalation into the cellular chromatin (for details see Materials and methods). The histograms represent the fluorescence intensity of PI for the indicated groups. (B) Flow cytometry cell cycle analysis revealed that the population of cells in the S and G2/M phase was remarkably decreased by Tc muc (*P*≤0.05). For determination of the cell cycle checkpoint regulators, cells were harvested after 3 days and whole-cell lysates were prepared for immunoblotting with specific atibodies against cyclin D3, p27^kip1^, and actin antibodies used to assure uniform loading (bottom row). Optical densitometry of the western blots used NIH Image software, where cyclin D3 and p27^kip1^ expression was normalized with the actin expression. (C) Expression of cyclin D3 was increased in anti-CD3 activated CD4^+^ T cells as compared to controls (*P≤*0.05); this increase was not observed when stimulation was done in the presence of Tc Muc (*P* = 0.0383); *the sialylation abolished the property of Tc Muc to induce downmodulation of cyclin D3 expression (*P = *0.0067). Expression of p27^kip1^ was decreased in anti-CD3 activated CD4^+^ T cells as compared to controls (*P≤*0.05); this decrease was not observed when stimulation was done in the presence of Tc Muc (*P*≤0.05); # the sialylation abolished the property of Tc Muc to induce upregulation of p27^kip1^ expression (*P≤*0.05). These data are representative of two independent experiments.

While several mechanisms are known to interfere with cell proliferation, they act in different cell cycle phases. We decided to analyze the effects of Tc Muc in specific cell cycle phases. Purified CD4^+^ T cells from naïve spleens were stimulated with plate bound anti-CD3 in the presence or absence of native *T. cruzi* mucins (20 µg/ml) for 72 hr and stained with the chromatin intercalating dye propidium iodide (PI) after nuclease digestion for analysis of the cell cycle by flow cytometry. Our data revealed 14% of CD4^+^ T cells in the M phase of the cell cycle when they were activated with anti-CD3 in the presence of Tc Muc, as compared with 42% of positive control cells activated with anti-CD3 only. This remarkable decrease was partially reverted upon neuraminidase treatment, as our findings indicate 33% of CD4^+^ T cells in M phase of the cell cycle when the cells were treated with desialylated Tc Muc during activation with anti-CD3 (Figure 5b). Further, an inverse correlation was found with the population of cells in the G1 phase, as we observed 45% of CD4^+^ T cells were in G1 phase of the cell cycle when they were activated with anti-CD3 in the presence of Tc Muc, as compared with 19% of positive control cells activated with anti-CD3 only (*P≤*0.05). Activation of CD4^+^ T cells in the presence of desialylated Tc Muc yielded 33% of the cells in G1 phase, showing a significant decrease (*P≤*0.05) of the cells in this phase as compared to the controls activated in the presence of the native Tc Muc (Figure 5b). These results indicate that Tc Muc inhibits cell proliferation by the induction of cell cycle arrest in G1 phase.

We next investigated the effects of Tc Muc on G1 cell cycle regulators, specifically cyclin D3 and the mitogen repressor p27^Kip1^ [45]–[47]. To evaluate the Tc Muc inhibitory effect on T lymphocyte activation, CD4^+^ T cells isolated from naive mice were stimulated with plate bound anti-CD3 mAb in the presence or absence of graded doses of native or desialylated Tc Muc. As demonstrated in Figure 5, activated CD4^+^ T cells show a typical profile of proliferating T cells, with upregulation of cyclin D3 and down-regulation of p27^Kip1^ (Figure 5b). In contrast, decreased cyclin D3 protein levels were associated with impaired TCR/CD3-triggered CD4^+^ T cell activation in the presence of Tc Muc for 72 h. This result was correlated with elevated expression of cell cycle repressor p27^Kip1^. Tc mucin did not affect the actin protein levels, which persisted throughout the 72 h experiment period of the T cells’ proliferation in response to anti-CD3 (Figure 5). Most interestingly, when CD4^+^ T cells were treated with desialylated Tc Muc during the CD3-activation protocol, we observed a reversion of the inhibitory profile as demonstrated by the upregulation of cyclin D3 and down-modulation of p27^Kip1^, a profile similar to what is described for CD3-activated T cells (Figure 5b). Based on our results, we postulated that Tc Muc might show potent antiproliferative effects on CD4^+^ T cells, inducing G1 phase arrest, by increasing the amount of p27^Kip1^ beyond a putative threshold.

### Triggering of the Sialic Acid-binding Ig-like Lectin-E Receptor (Siglec-E) Induce Supression of CD4^+^ T Cell

**Figure 6:**
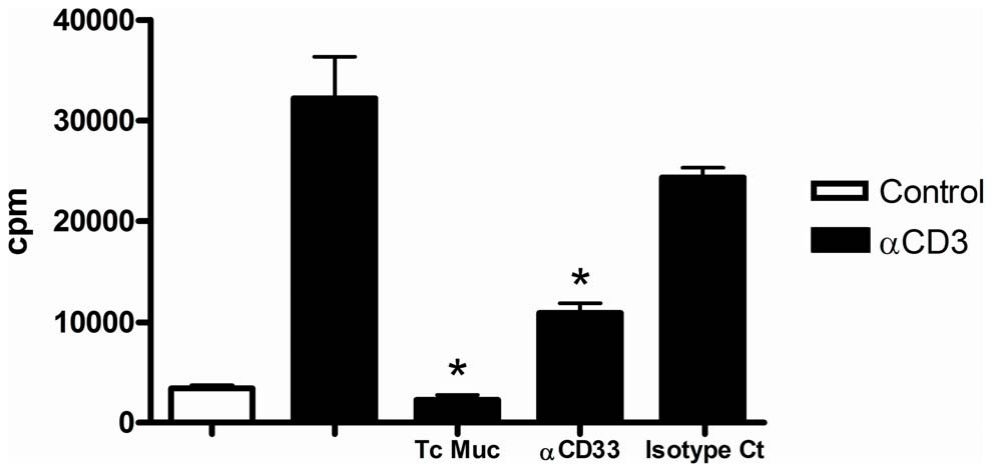
Triggering of the Tc Muc ligand Siglect E (CD33) induces suppression of CD4^+^ T cell proliferation *in vitro*. Purified CD4^+^ T cells from naïve spleens were stimulated with plate bound anti-CD3 for 72 hr, in the presence or absence of anti-CD33 or isotype control antibody (40 µg/mL). Proliferation was measured 72 h after stimulation by [^3^H]thymidine incorporation. *Differences between Tc mucin or anti-CD33 treatment *versus* anti-CD3 stimulated positive control are significant (*P*≤0.05).

The interaction mediated by glycoconjugates expressed on parasites and sialic acid-binding Ig-like lectin-E expressed on the host cells may account for the effects in a complex and dynamic situation at the interface between parasites and host cells. In the present work, we investigated whether the Siglec-E could mediate suppression of CD4^+^ T cells. To address this question, equal numbers of purified CD4^+^ T cells isolated from naive mice were stimulated by plastic-absorbed anti-CD3 mAbs in the presence or absence of Tc Muc anti-Siglec-E (CD33) or isotype control antibody. As we expected, CD4^+^ T cell population show the ability to respond to plastic-coated anti-CD3 mAbs and this expansion was abrogated when the cells were cultured in the presence of Tc Muc. Most interestingly, when CD4^+^ T cells were co-cultured with plastic-coated anti-CD3 mAb together with anti-CD33 but not isotype control antibody, we observed a statistically significant abrogation of the proliferative CD4^+^ T cell response (Figure 6).

### Inhibition of *in vivo* Development of CD4^+^ T Cell Responses in *Trypanosoma cruzi* Infection

**Figure 7:**
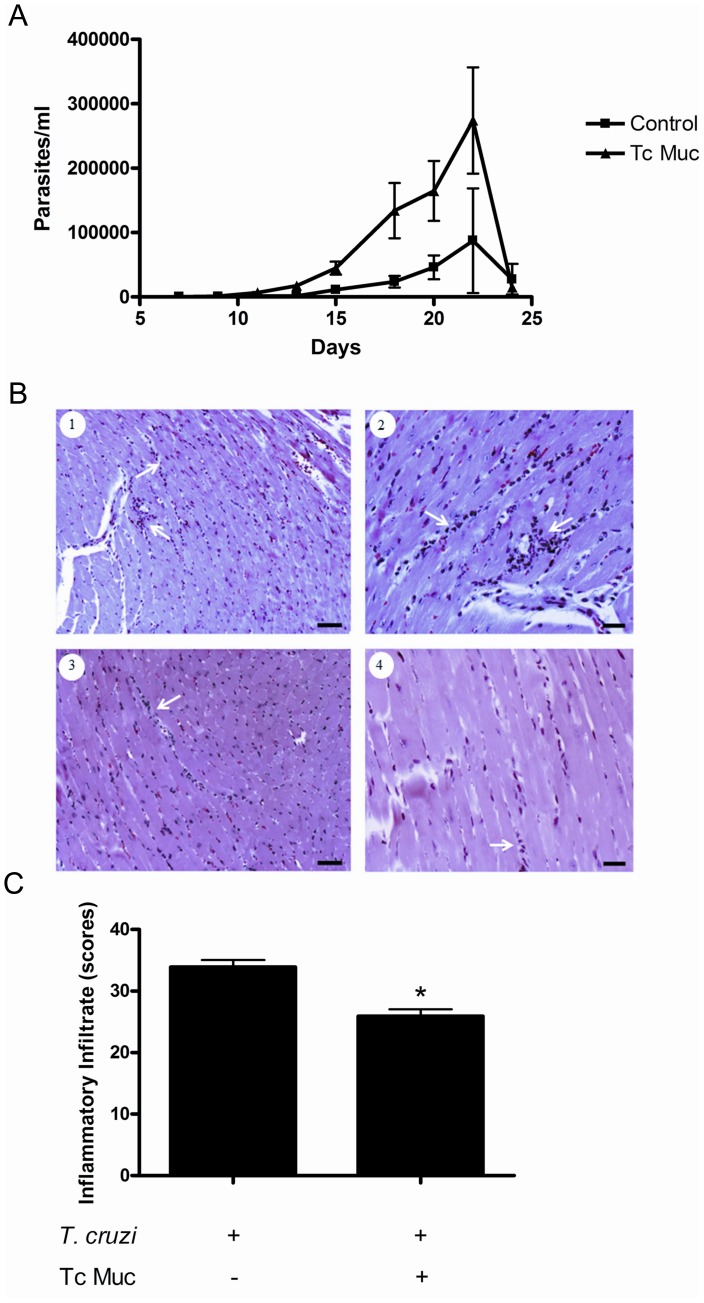
*T. cruzi* infected mice develop a higher parasitemia and reduced heart inflammatory infiltration when treated with Tc Muc. BALB/c mice were infected via *i.p.* with 2×10^5^ chemically induced metacyclic forms of *Trypanosoma cruzi* Dm28c clone. The mice received *i.p.* injections of Tc Muc (20 µg/mouse) or PBS on alternate days starting at day of infection. (A) The parasitemia for each mouse group was represented as the mean ± the standard deviation (SD) (*n* = 5). The parasitemia of mice from the Tc Muc treated group was significantly higther than untreated control mice (*P*≤0.01). (B and C) Inflammatory infiltration is diminished in the heart by treatment with Tc Muc. Twenty four days after infection cardiac fragments were extracted from *Trypanozoma cruzi* infected mice (B, 1–2) untreated or (B, 3–4) treated with Tc Muc. Slides were stained with haematoxylin-eosin and cellular nuclei from inflammatory and resident cells counted using Leica QWin program in sections with different magnifications, 40× (B, left panels) and 100× (B, right panels). (C) Inflammatory indexes were determined by counting inflammatory foci (average counts per field). Data were obtained from survivors (2 independent experiments) and shown as mean/standard error of the mean. Asterisk (*P*≤0.05) means statistical difference between infected mice treated with Tc mucin *versus* infection control group.

To determine the *in vivo* effects of administration of Tc mucin, we injected intraperitoneally BALB/c mice with chemically induced metacyclic trypomastigotes from *T. cruzi* Dm28c strain (2×10^5^). The data shown in Figure 7a indicate that control mice infected with TCT developed a low blood parasitemia. In contrast, infected mice treated with Tc mucin showed a precocious blood parasitemia at day 13 post-infection, which further increased (aproximately 3-fold) as the infection continued. The susceptibility of the Tc treatment was correlated with augmented infiltration of leucocytes in the heart at day 21 post-infection (Figure 7b–c).

**Figure 8:**
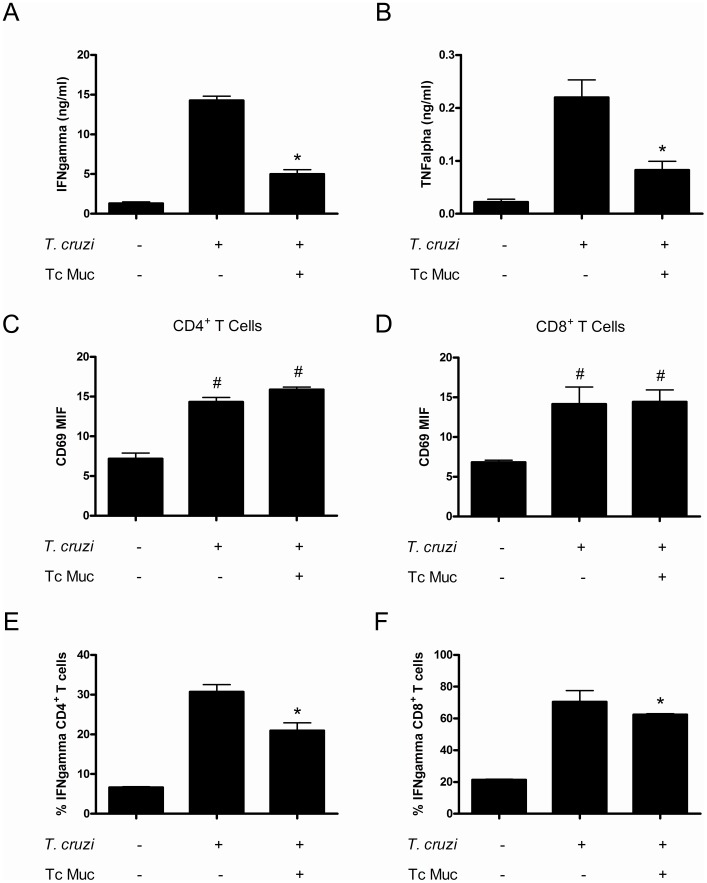
Splenocytes from *T. cruzi* infected mice treated with Tc mucin produce low levels of IFN-γ. BALB/c mice were infected *i.p.* with 2×10^5^ chemically induced metacyclic forms of *Trypanosoma cruzi* Dm28c clone. The mice received *i.p.* injections of Tc Muc (20 µg/mouse) or PBS on alternate days starting at day of infection. Non-infected mice were used as control group. Twenty four days after infection, purified splenocytes were stimulated with PMA and ionomycin, as described in the Methods section, and supernatants were harvested at 24 h for determination of (**A**) IFN-γ and (**B**) TNFα by ELISA. The *y*-axis represents the levels of cytokines, detected by a specific ELISA assay, expressed in ng/ml. Asterisks represent statistical significance (p<0.05) as determined by the Student t test. To access the T cell activation profile CD69 expression on both CD4^+^ (**D**) and CD8^+^ (**E**) T cells were analysed by FACS analysis; the frequency of IFN-γ producing T cells from splenocytes polyclonally stimulated with PMA/ionomycin, and the percentages of both IFN-γ producing CD4^+^ T cells (**F**) and CD8^+^ T cells (**G**)**,** were determined by intracellular cytokine FACS-staining. #Infected group are statistically different from non-infected control mice (*P*≤0.05). Asterisks represent statistical differences between Tc mucin treated *versus* non-treated mice from infected groups as determined by the Student t test (*P*≤0.05). All experiments were repeated at least 3 times.

Since the protective responses againt *T. cruzi* infection are associated with the development of IFN-γ dependent responses, we set out to determine any difference in the levels of type-1 effector T cells. Recall assays upon polyclonal stimulation showed that IFN-γ production by splenic cells from Tc mucin-treated mice was significantly diminished (over 50%) as compared to responses elicited by experienced splenocytes isolated from infected control mice at 21 d.p.i. (Figure 8a). These findings were also correlated with reduced levels of TNF-α production by splenic cells from Tc mucin-treated mice under polyclonal stimulation, indicating that type 1 protective responses could be affected in those mice (Figure 8b). Since our findings demonstrated that the *Trypanosoma cruzi* sialoglycoproteins can modulate the splenic cytokine response, a matter that could be related to the enhanced parasite virulence seen in the *in vivo* administration of mice with Tc mucin, we next tested the hypothesis that the infection in Tc Muc-treated mice may have altered the T cell responses, thus inducing a loss of protection of parasitic load and increased signs of disease. Since the protective responses againt *T. cruzi* infection are associated with the development of IFN-γ dependent responses, we set out to determine whether the *Trypanosoma cruzi* sialoglycoproteins can modulate the T cell activation profile, a matter that could be related to the enhanced parasite virulence seen in the *in vivo* administration of mice with Tc mucin. Using multiparameter FACS analysis we assessed the expression of the major cell surface markers that are known to undergo changes after *in vivo* activation of T cells. We found that both CD4^+^ and CD8^+^ T cells from *T. cruzi* infected animals treated with Tc mucin express the CD69 marker at levels comparable to the infected controls (Figures 8c–d). However, recall assays upon polyclonal stimulation showed a negligible reduction of frequency of both IFN-γ secreting CD4^+^ T cells and CD8^+^ T cells from Tc mucin-treated mice in comparison with the control infected group, indicating that type 1 protective responses could be affected in those mice (Figures 8e–f). Collectively, these results suggest that the inhibitory role of Tc mucin has an impact on the control mechanisms affecting the host’s protective cellular responses during *T. cruzi* infection.

## Discussion

The relative strengths of the host immune system and pathogen virulence influence the lifespan of the infection, and pathogens have evolved many strategies to evade the immune system of the host and establish chronic infections. *T. cruzi* provides a good example of such a strategy: its surface is covered by sialic acid residues and it produces a unique enzyme, the *trans*-sialidase that transfers sialic acid from host glycoconjugates to mucin-like molecules on its surface [27]–[30]. *In vitro* studies have shown that these mucins are the most abundant glycoproteins on the surface of the parasite and that they play a key role in how the parasite invades the host and avoids its immune system [14]–[16], [21]–[23]. The sialylated mucins mask parasite antigenic determinants, so protecting the parasite from host attack by, for example, antigalactosyl antibodies and complement factor B [23], [48]–[50].

Interestingly, recent studies have demonstrated that in addition to masking parasite antigens, the *T. cruzi* surface sialic acid is also responsible for direct interaction with the inhibitory host cell receptor, Siglec-E [33], [37]. Moreover, ligation of Siglec-E on the DC surface with cross-linking antibodies reduces the capacity of T cells to be activated and proliferate [33]. Furthermore, it has been shown that binding of Tc Muc to Siglec-9 results in a dampening of cell function and is related to the production of IL-10 [33]. Mucins derived from other organisms and species, such as mammals, can also inhibit T cell proliferative responses. In recent years, it has also been shown that the inefficient host immune response to cancer antigens is at least in part due to the presence of carcinoma-associated mucins [51]–[55].

In the present study, we have presented evidence that Tc mucin is able to inhibit CD4^+^ T cell proliferation. The supression of T cell responses has been shown to be a result of the diminished production of IL-2 and its receptor CD25 [14]–[16]. In fact, we found that exposure to Tc Muc significantly diminished the level of IL-2 secretion in response to TCR activation. Moreover, our data show that the T cell anergy induced by Tc mucin was not reversed by exogenous IL-2, indicating that the IL-2 pathway is impared when CD4^+^ T cells are activated in the presence of Tc Muc. To further analyze the effects of Tc Muc treatment, we tested whether cytokine secretion was affected. According to our findings, Tc Muc was able to inhibit the production of IFN-γ, TNF-α, IL-2, IL-4, IL-10 and TGF-β cytokines by TCR-stimulated CD4^+^ T cells. This inhibitory property of Tc Muc may affect the course of the parasite-host interaction during the acquisition of cell-mediated adaptive immune responses, therefore damping protective host responses and so establishing persistent infections.

Our findings indicating that Tc Muc has such a strong inhibitory effect on T lymphocytes is in agreement with experiments showing that the *T. cruzi*–associated mucins have an immunosuppressive effect [14]–[16]. It is also consistent with clinical observations that host animals acutely infected with *T. cruzi* develop symptoms of immunosuppression, including functional alterations of lymphocytes and other cells involved in immune responses [56]–[64]. The sialylated ligands are strong candidates to interfere with host immunological responses, both innate and adaptive [38]. In this connection, it has been suggested that interactions involving these ligands alter leucocyte function and thereby facilitate the establishment of infection. It was shown recently that the interaction of Siglec-9 with sialylated ligands produced by *Streptococcus* reduced neutrophil responses and increased survival of the bacteria [65]. We have used anti-Siglec E antibodies to examine whether the cross-linking of surface Siglec-E inhibits T cell proliferation. We found that mab concentrations of up to 5 µg/ml did significantly inhibit the proliferation of stimulated T cells.

In the light of this finding, we propose that the T cell surface mucin receptor Siglec-E is implicated in the inhibition of T cell proliferation. Importantly, our results showing induction of G1 cell cycle arrest associated with up-regulation of the cyclin D inhibitor p27 on activated CD4^+^ T cells further support a modulatory role of sialylated Tc Muc in signal transduction during T cell activation. The p27 is a phosphatase regulator that appears to participate in the G1 cell cycle arrest checkpoint [45]–[47]. The observation that desialylated Tc Muc loses its anti-mitogenic effect strengthens this notion. As the desialylated Tc Muc still contain a high content of O-linked oligosaccharides it is possible that its remaining T cell inhibitory effects after neuraminidase treatment could be due to the binding of O-glycan moieties to other host lectin receptors. Importantly, we found that exposure of mice to Tc Muc when they were being infected with *Trypanosoma cruzi* increased their susceptibility to infection as shown by increased parasitemia and heart damage at 21 dpi. At the same time we noted a lower frequency of IFN-γ producing CD4^+^ and CD8^+^ T cell responses correlated with decreased levels of both splenic IFN-γ and TNF-α cytokines in mice treated with Tc mucin.

In conclusion, we have accumulated evidence that *T. cruzi* mucins are involved in T cell responses by affecting the proliferation of T cells. It is likely that Siglec-E is involved in this effect, as we showed that activation of this receptor also inhibits the T cell proliferative responses. Further studies are required to elucidate the intricate intracellular mechanisms which link *T. cruzi* mucins with other T cell surface and intracellular regulators. Although cross-linking of Siglecs by antibodies is a useful tool for dissecting the function of these molecules, use of Siglec-E deficient mice in future could permit more detailed insight into the consequences of *T. cruzi* infection. This could help us to understand how the *T. cruzi* derived-mucin glycoconjugates influence immune responses.

## Supporting Information

Figure S1 **Tc mucin inhibits Thy-1 triggered CD4** ^+^ **T cell proliferation.** Purified CD4^+^ T cells from naïve spleens were stimulated with plate bound anti-CD3 for 72 hr, in the presence or absence of 50 µg/ml Tc Muc. Proliferation was measured 72 h after stimulation by [^3^H]thymidine incorporation. *Differences between Tc Muc *versus* anti-Thy1.1 treatment are significant (*P*≤0.0001). The inhibition of proliferation by Tc Muc was not observed when control mucin derived from bovine submaxillary glands was used (50 µg/ml). Results are the means ±SD of triplicate cultures of three different experiments.
(TIF)

Figure S2 **Measurement of T cell viability in the presence of Tc muc in a dose-dependent manner.** Total splenic T cells seeded at 100 µL/well in a flat-bottom 96-well plate (3×10^5^ cells/well) were cultured in DMEM supplemented with 10% FBS in the presence of various doses of Tc Muc. Cell viability was measured by adding 3-[4,5-dimethylthia-zol-2-yl]-2,5-diphenyltetrazolium bromide (MTT) assay at a 1/10 volume of the total cell culture volume at 18 hr of culture. After incubating for 4 hours, 0.01 N HCI with 10% sodium dodecyl sulfate was added (100 µL/well) to dissolve the formazan crystals formed by live cells, and the absorption of each well was measured by an enzyme-linked immunosorbent assay plate reader (Molecular Devices Co., Sunnyvale, CA, USA) at 540 nm. Values represent the mean ± SD absorbance of triplicate cultures.
(TIF)

Figure S3 **Carbohydrate analysis and correlation spectroscopy of the T. cruzi Dm28c strain sialoglycoproteins.** Intact siloglycoproteins were methanolized with 0.5 M HCl in methanol for 18 h at 80°C, neutralized with silver carbonate and re-N-acetylated with acetic anhydride. The dried residue was trimethylsilylated by addition of bis(trimethylsilyl)-trifluoro-acetamide/pyridine (1∶1 v/v). The products were analyzed by gas-liquid chromatography (GC) on a DB-1 fused silica column (30 m×0.25 mm i.d.) using hydrogen as the carrier gas. The column temperature was programmed from 120 to 240°C at 2°C min^−1^. **(A)** Monosacccharide analysis by GC of the trimethylsilylated methylglycosides demonstrating the presence of (1) Man; (2) Gal; (3) GlcNAc and (4) Neu5Ac in a molar ratio of 3∶1.5∶1∶0.5. Electron impact-mass spectrum of per-O-trimethylsilylated Neu5Ac (4). Insert: 15% SDS–PAGE of siloglycoproteins from T. cruzi Dm28c strain and stained with periodic acid/Schiff’s reagents for carbohydrate detection. **(B)** Partial 600 MHz TOCSY spectra of sialoglycoproteins purified from T. cruzi Dm28c strain. The spectra were obtained at 25°C, using an 80-ms mixing time. The spectral regions are numbered as follows: 1, GlcNAcβ1→NAsn H-1 trace; 2, cross-peaks arising from β-Galp residues attached to the GlcNAcα1→OThr; 3, cross-peaks arising from correlations between the Neu5Ac H-3eq and ring protons.
(TIF)

Figure S4 **Effect of neuraminidase-treatment on T. cruzi mucin.** Western blot following non-reducing SDS gel electrophoresis showing the effect of incubation of Tc Muc with 0.2 U/mL of *V. cholerae* neuraminidase on Maackia amurensis (MAA) binding. MAA binding to Tc Muc corroborates the presence of sialic acid–2→3Gal (Line A). Neuraminidase treatment of Tc Muc abrogated staining by MAA (Line B). Protein load to the gel was detected by silver staining (Bottom line). Purified Tc Muc (1 µg) was electrophoresed on 10% SDS-PAGE gels and blotted onto nitrocellulose membranes. The membrane was blocked in a blocking solution (150 mM NaCl, 10 mM Tris, pH 7.5, 10% Tween 20) for 2 h at room temperature. The membranes were incubated for 1 h with 10 µg/ml biotin-labeled Maackia amurensis lectin (EY Laboratory). Membrane was washed five times and incubated with a 1∶2000 dilution of anti-biotin horseradish peroxidase conjugate (Cell Signaling Technology) for 60 min, and the reaction was developed with SuperSignal West Pico chemiluminescence reagents (Pierce).
(TIF)
