# Supplementary figures and images for: Inhibitory Effects of Trypanosoma cruzi Sialoglycoproteins on CD4+ T Cells Are Associated with Increased Susceptibility to Infection
Source: PLoS One. 2013 Oct 28;8(10):e77568. doi: 10.1371/journal.pone.0077568 (PMC3810146; doi:10.1371/journal.pone.0077568)

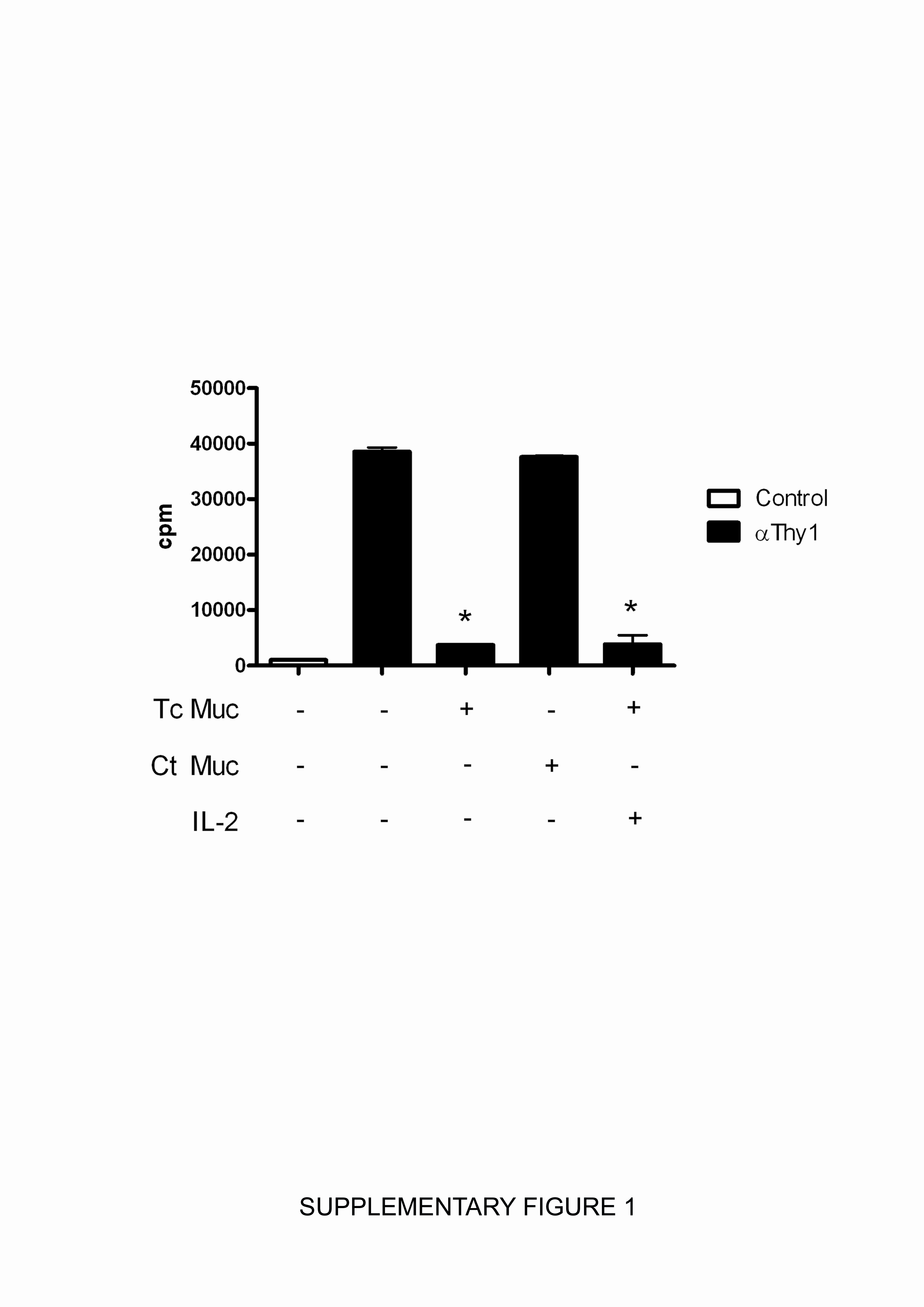

Supplement: Figure S1 — Tc mucin inhibits Thy-1 triggered CD4 + T cell proliferation. Purified CD4+ T cells from naïve spleens were stimulated with plate bound anti-CD3 for 72 hr, in the presence or absence of 50 µg/ml Tc Muc. Proliferation was measured 72 h after stimulation by [3H]thymidine incorporation. *Differences between Tc Muc versus anti-Thy1.1 treatment are significant (P≤0.0001). The inhibition of proliferation by Tc Muc was not observed when control mucin derived from bovine submaxillary glands was used (50 µg/ml). Results are the means ±SD of triplicate cultures of three different experiments. (TIF) [file pone.0077568.s001.tif]

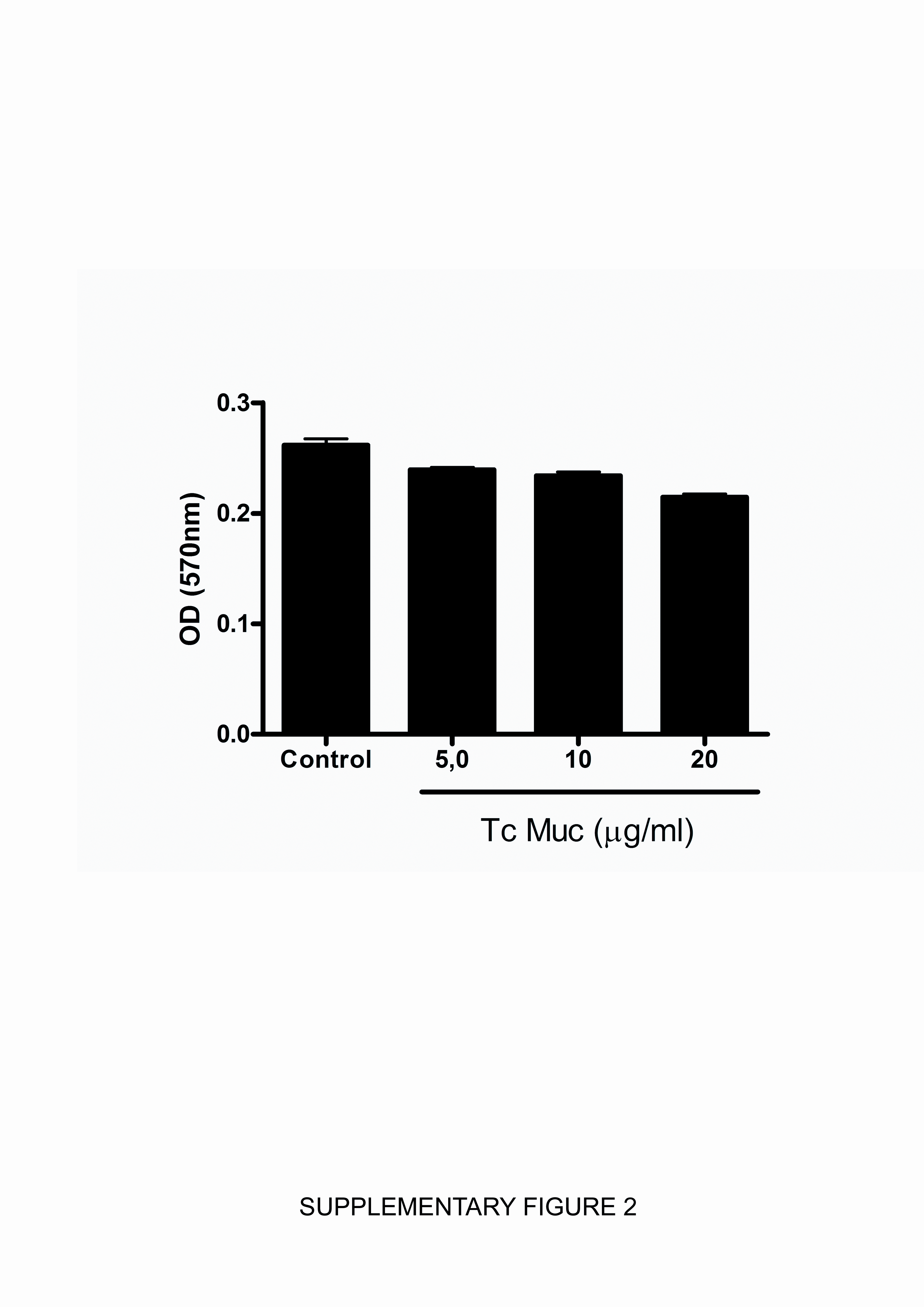

Supplement: Figure S2 — Measurement of T cell viability in the presence of Tc muc in a dose-dependent manner. Total splenic T cells seeded at 100 µL/well in a flat-bottom 96-well plate (3×10^5^ cells/well) were cultured in DMEM supplemented with 10% FBS in the presence of various doses of Tc Muc. Cell viability was measured by adding 3-[4,5-dimethylthia-zol-2-yl]-2,5-diphenyltetrazolium bromide (MTT) assay at a 1/10 volume of the total cell culture volume at 18 hr of culture. After incubating for 4 hours, 0.01 N HCI with 10% sodium dodecyl sulfate was added (100 µL/well) to dissolve the formazan crystals formed by live cells, and the absorption of each well was measured by an enzyme-linked immunosorbent assay plate reader (Molecular Devices Co., Sunnyvale, CA, USA) at 540 nm. Values represent the mean ± SD absorbance of triplicate cultures. (TIF) [file pone.0077568.s002.tif]

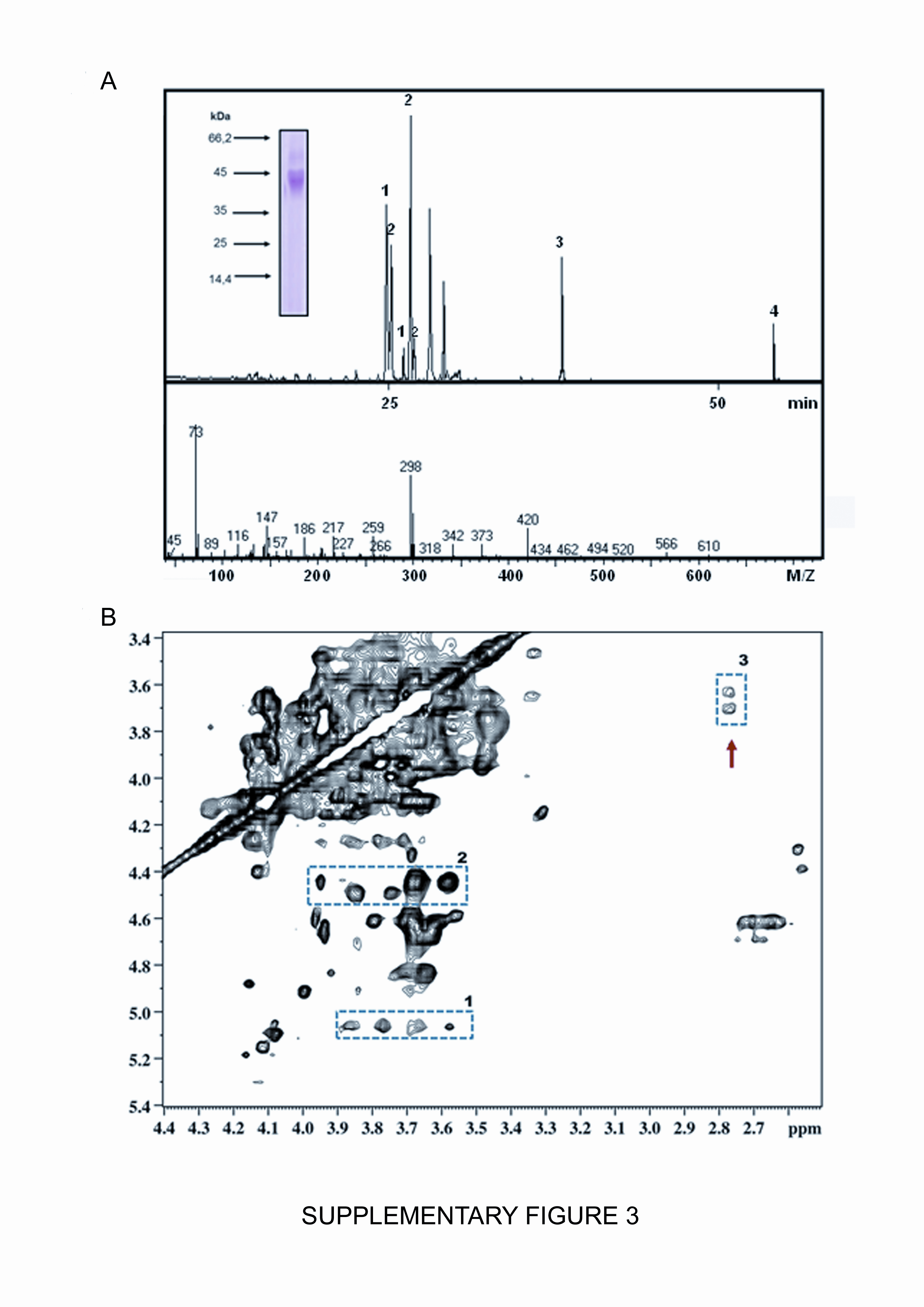

Supplement: Figure S3 — Carbohydrate analysis and correlation spectroscopy of the T. cruzi Dm28c strain sialoglycoproteins. Intact siloglycoproteins were methanolized with 0.5 M HCl in methanol for 18 h at 80°C, neutralized with silver carbonate and re-N-acetylated with acetic anhydride. The dried residue was trimethylsilylated by addition of bis(trimethylsilyl)-trifluoro-acetamide/pyridine (1∶1 v/v). The products were analyzed by gas-liquid chromatography (GC) on a DB-1 fused silica column (30 m×0.25 mm i.d.) using hydrogen as the carrier gas. The column temperature was programmed from 120 to 240°C at 2°C min−1. (A) Monosacccharide analysis by GC of the trimethylsilylated methylglycosides demonstrating the presence of (1) Man; (2) Gal; (3) GlcNAc and (4) Neu5Ac in a molar ratio of 3∶1.5∶1∶0.5. Electron impact-mass spectrum of per-O-trimethylsilylated Neu5Ac (4). Insert: 15% SDS–PAGE of siloglycoproteins from T. cruzi Dm28c strain and stained with periodic acid/Schiff’s reagents for carbohydrate detection. (B) Partial 600 MHz TOCSY spectra of sialoglycoproteins purified from T. cruzi Dm28c strain. The spectra were obtained at 25°C, using an 80-ms mixing time. The spectral regions are numbered as follows: 1, GlcNAcβ1→NAsn H-1 trace; 2, cross-peaks arising from β-Galp residues attached to the GlcNAcα1→OThr; 3, cross-peaks arising from correlations between the Neu5Ac H-3eq and ring protons. (TIF) [file pone.0077568.s003.tif]

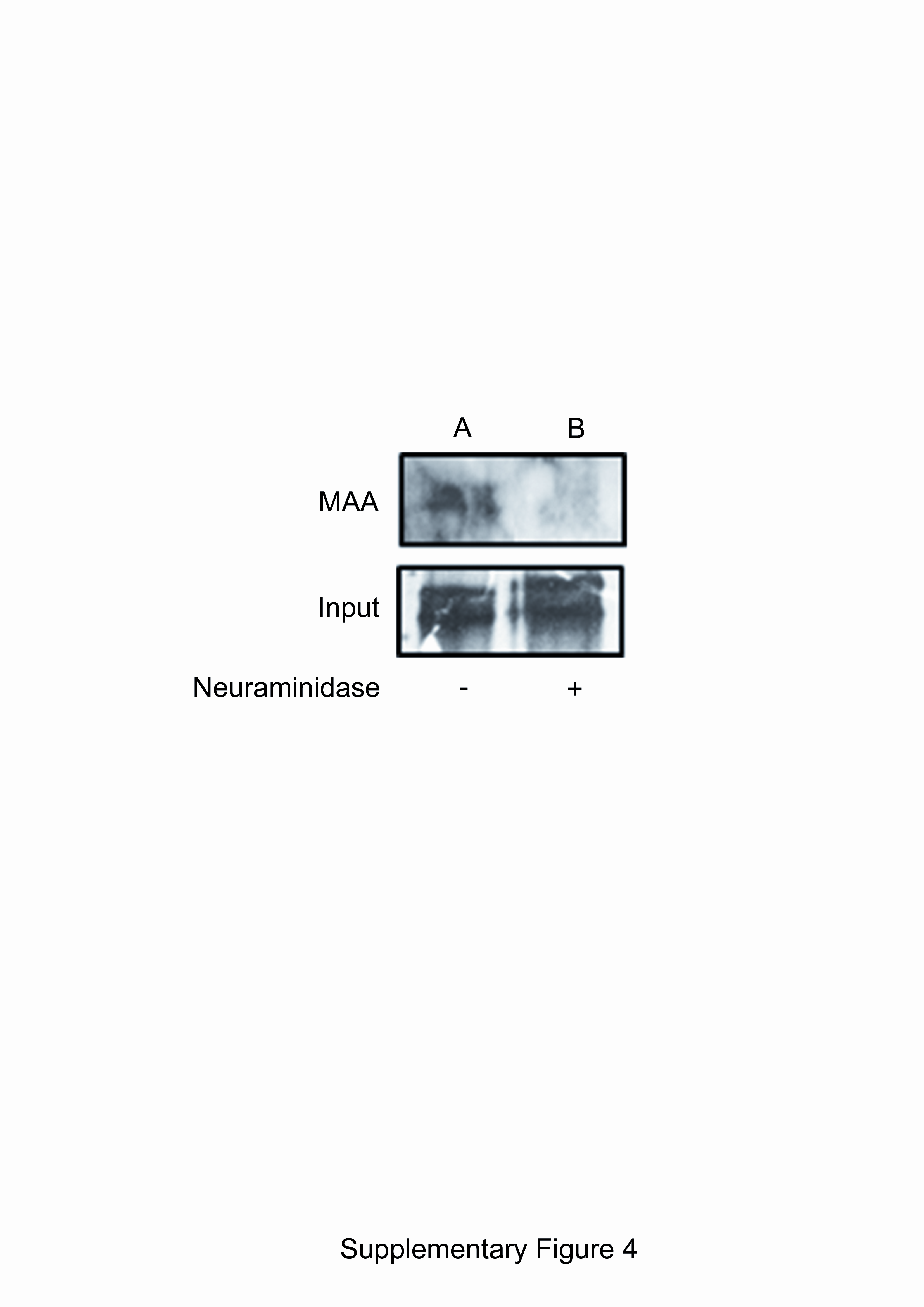

Supplement: Figure S4 — Effect of neuraminidase-treatment on T. cruzi mucin. Western blot following non-reducing SDS gel electrophoresis showing the effect of incubation of Tc Muc with 0.2 U/mL of V. cholerae neuraminidase on Maackia amurensis (MAA) binding. MAA binding to Tc Muc corroborates the presence of sialic acid–2→3Gal (Line A). Neuraminidase treatment of Tc Muc abrogated staining by MAA (Line B). Protein load to the gel was detected by silver staining (Bottom line). Purified Tc Muc (1 µg) was electrophoresed on 10% SDS-PAGE gels and blotted onto nitrocellulose membranes. The membrane was blocked in a blocking solution (150 mM NaCl, 10 mM Tris, pH 7.5, 10% Tween 20) for 2 h at room temperature. The membranes were incubated for 1 h with 10 µg/ml biotin-labeled Maackia amurensis lectin (EY Laboratory). Membrane was washed five times and incubated with a 1∶2000 dilution of anti-biotin horseradish peroxidase conjugate (Cell Signaling Technology) for 60 min, and the reaction was developed with SuperSignal West Pico chemiluminescence reagents (Pierce). (TIF) [file pone.0077568.s004.tif]
